# Reconstructing Aesthetics: *Aesthetic Surgery Journal* and Aesthetic Breast Reconstruction

**DOI:** 10.1093/asj/sjag041

**Published:** 2026-09-16

**Authors:** Maurice Y Nahabedian, Nolan Karp

Welcome to this special Aesthetic Breast Reconstruction issue of *Aesthetic Surgery Journal* (*ASJ*) in celebration of its 30th anniversary. In this editorial, we will review the various innovations and advancements, as well as the evolution of breast reconstruction and how it has become an aesthetic operation.

## AESTHETIC BREAST RECONSTRUCTION: A BRIEF HISTORY

Surgical breast reconstruction dates back to the late 19th century, with the first documented instance being performed in Germany by Vincent Czerny in 1895, followed in 1905 by the first breast reconstruction using the pectoral muscle to form a mound.^1,2^ It is no longer acceptable to simply create a mound on the chest as it was during the first half of the 20th century, the early years of reconstruction. With the invention of the silicone gel breast implant in 1963, surgeons from the 1970s onward began to expand what was possible for breast surgery.^3-5^ These crucial developments allowed breast surgeons to perform surgeries that were not only lifesaving but could also achieve a beautiful aesthetic result. Today's patients expect, and surgeons can deliver, outcomes that are far superior to what was achievable 50 years ago. When we reflect on our personal experiences from the 1980s and 1990s to now, it is astounding how our techniques and strategies have evolved. What we learned about breast reconstruction during our residencies serves as a foundation for what we do today, as so much has changed for the better and translated into fewer complications, improved aesthetic outcomes, and higher patient satisfaction.

## AESTHETIC BREAST RECONSTRUCTION AND *AESTHETIC SURGERY JOURNAL*

**Figure 1.**
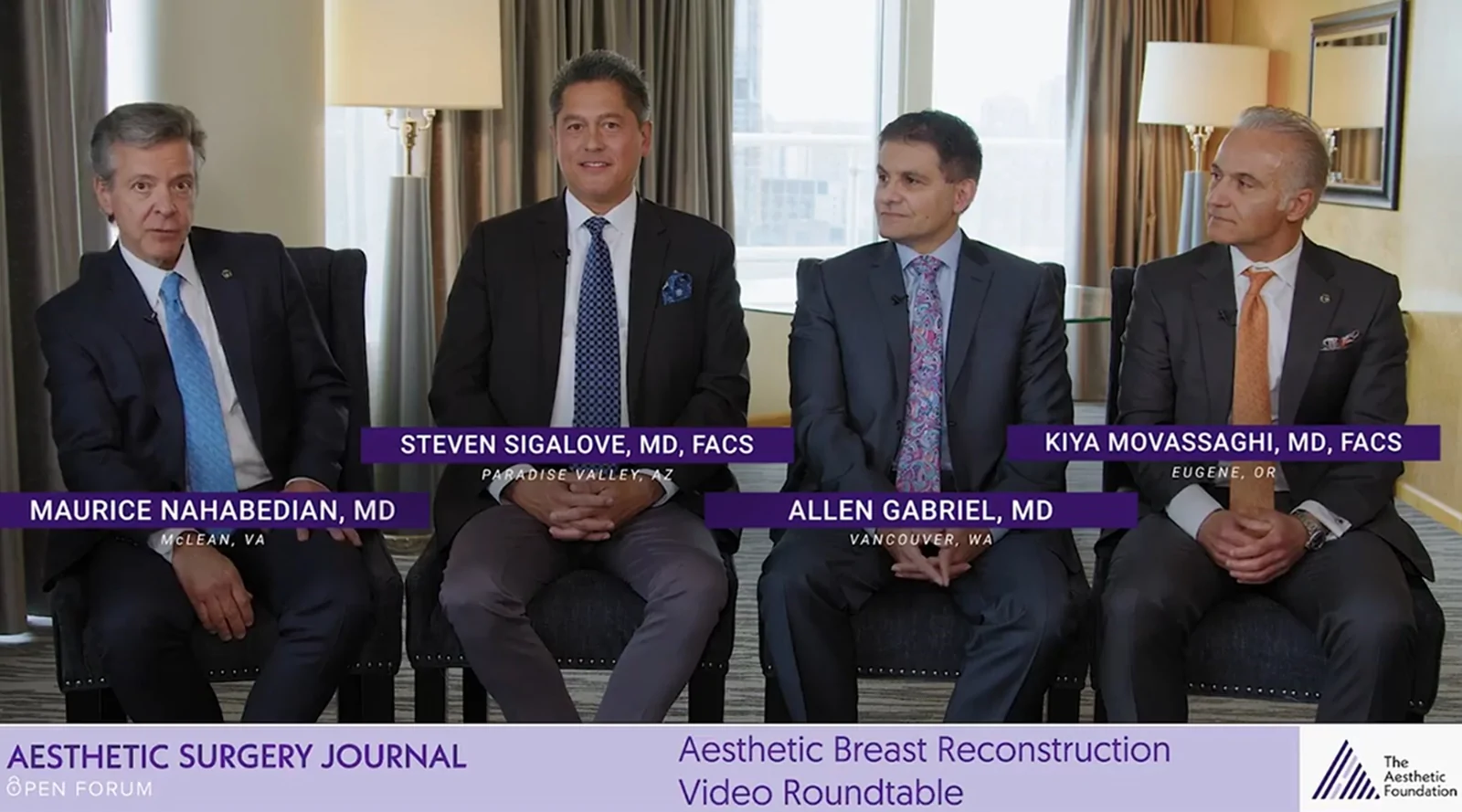
*Aesthetic Surgery Journal* Aesthetic Breast Reconstruction section editor Dr Maurice Nahabedian (far left) moderating the “Aesthetic Breast Reconstruction: An Expert Video Roundtable Discussion,” with discussants Dr Steven Sigalove (left), Dr Allen Gabriel (middle), and Dr Kiya Movassaghi (right), as published in *ASJ Open Forum* and sponsored by The Aesthetic Foundation at The Aesthetic Meeting 2024 in Vancouver, Canada.^7^.

The Aesthetic Breast Reconstruction section was launched in 2023.^6^ Our goal was to make this section unique and different from other journals in that its purpose was to emphasize the aesthetic aspects of breast reconstruction. Since its launch, the section has seen over 80 articles published (and counting!), as well as many articles published in *ASJ Open Forum* (Figure 1).^7^ Today's patients expect outcomes that are natural and aesthetically pleasing, and many want to improve upon the appearance of their breasts. As such, plastic surgeons have sought various innovative strategies intended to improve surgical outcomes and reduce complications. This has been achieved by applying various principles and concepts unique to plastic surgery that have evolved over the decades. Many of these current innovations and advancements are captured within this section and have served to benefit our global readership.

**Figure 2.**
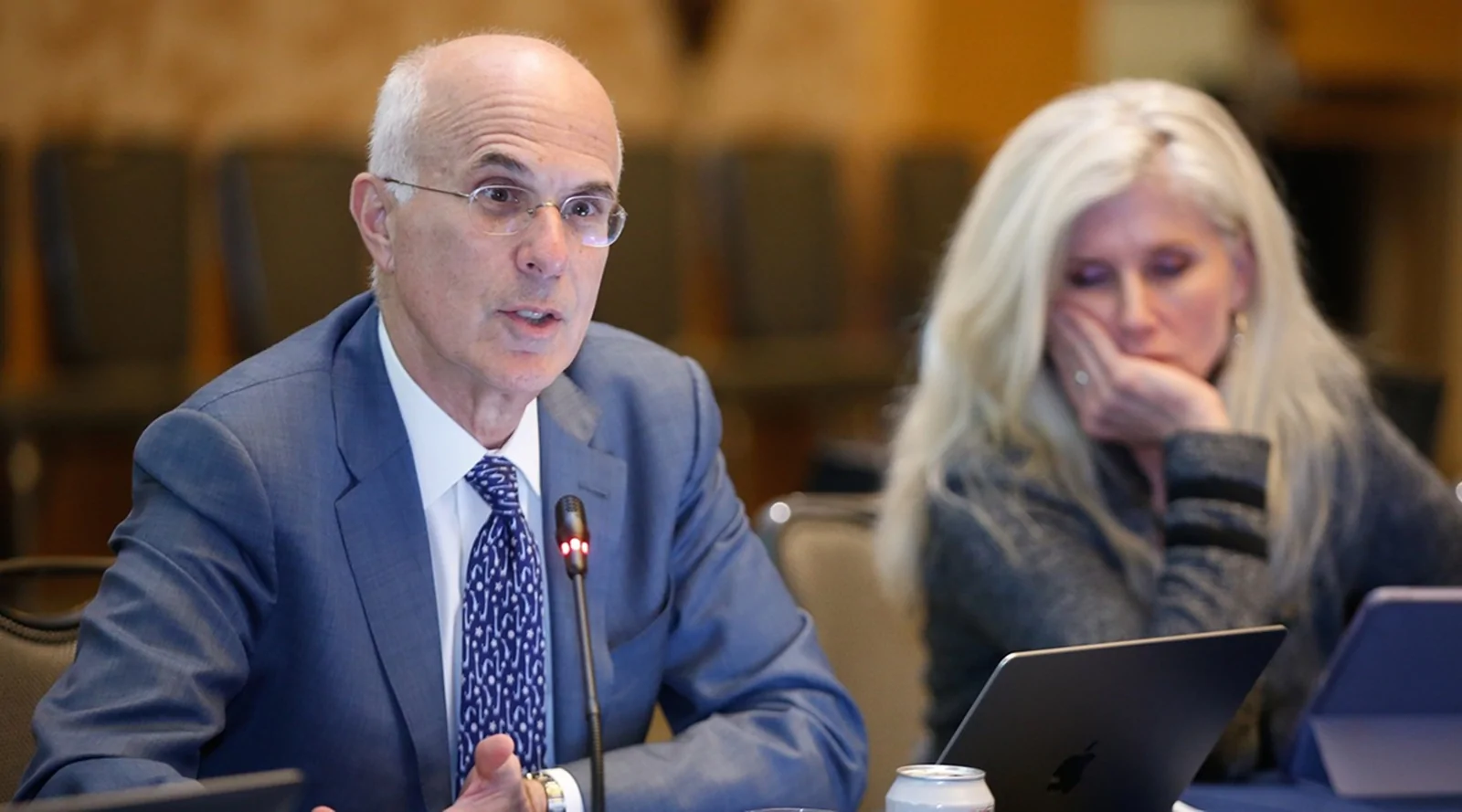
Dr Nolan Karp (left) and Dr Melinda Haws (right) photographed together at The Aesthetic Meeting 2022 in Vancouver, Canada.

**Figure 3.**
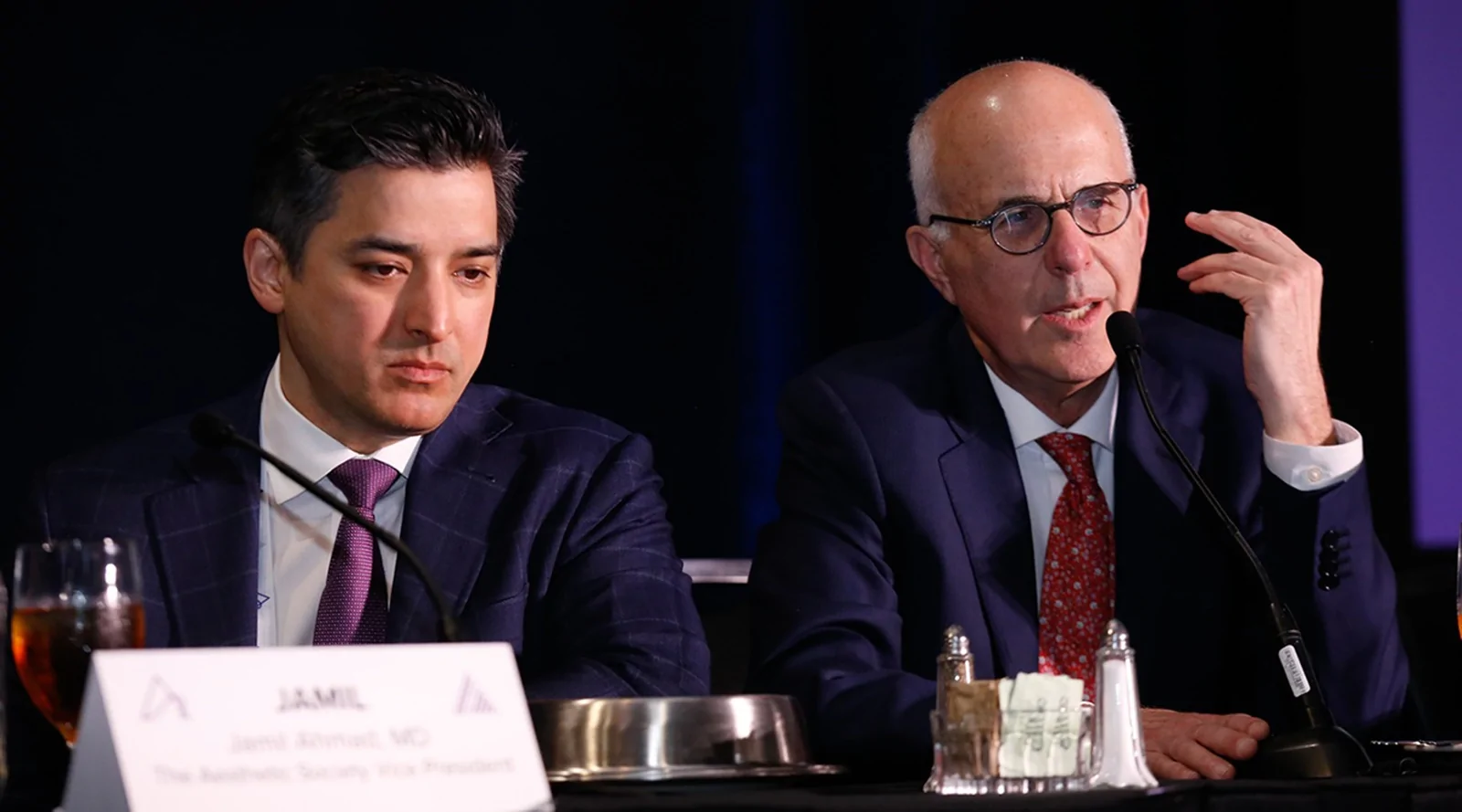
*Aesthetic Surgery Journal* (*ASJ*) Aesthetic Breast Reconstruction section editor Dr Nolan Karp (right) and *ASJ* Body Contouring section editor Dr Jamil Ahmad (left) participating in a panel discussion at The Aesthetic MEET 2025 in Austin, TX.

Plastic surgeons are inherently interested in “aesthetics” and optimizing outcomes following all forms of breast surgery, and this section has provided a forum for just that. It is this merging of patient expectation and surgical technique that was our primary goal for this section. *ASJ* has prided itself on providing its readership with strategies for success that are focused on aesthetic optimization for breast, body, and facial procedures. The combination of reconstructive principles and aesthetic concepts was the impetus for initiating this section and is the basis from which we will continue to evolve. We feel that it is important for breast reconstruction to be included in this aesthetic arena because all patients, aesthetic and reconstructive, are inherently interested in natural and pleasing outcomes. Thus, we have encouraged authors to submit manuscripts that are focused on all forms of breast reconstruction that include autologous, prosthetic, and oncoplastic. Plastic surgeons understand that all patients are different and that there is no single technique or strategy that can be applied to everyone, which is reflected by the diversity of thought, discipline, and approach that can be found in the pages of *ASJ* and in the work of the dedicated members of The Aesthetic Society who perform these procedures (Figures 2, 3).

As we reflect on some of the aesthetic breast reconstruction manuscripts published in *ASJ*, there are several that come to mind that emphasize these aesthetic principles and concepts. The first paper is focused on autologous reconstruction, titled “Breast Reconstruction with Simultaneous Bilateral Lumbar Artery Perforator Flaps Improves Waistline Definition and Buttock Projection.”^8^ The authors evaluated 50 patients and demonstrated through digital morphometry that after reconstruction, patients were found to have a more defined waistline and a more projected buttock. Patients were surveyed postoperatively, and 73% indicated that their waistline had aesthetically improved and 53% reported an aesthetic improvement in the buttock. The second manuscript is “Implant-Based Breast Reconstruction after Nipple-Sparing and Skin-Sparing Mastectomy in Breast-Augmented Patients.”^9^ These patients often pose unique challenges based on having certain aesthetic ideals and expectations and then having to undergo mastectomy because of cancer. The authors demonstrated that immediate prepectoral implant reconstruction represents a safe, viable, and aesthetically pleasing option for patients following mastectomy who have undergone previous breast augmentation. The final manuscript is titled “Head-to-Head Analysis of Vertical vs Horizontal Incision Patterns in Breast Reconstruction.”^10^ The authors were able to demonstrate that there were no differences in the complication rate following one incision or the other; however, they did demonstrate that the vertical incisions were preferred aesthetically in a crowdsourcing analysis of the general population. In addition to these 3 standout articles, the Aesthetic Breast Reconstruction section of *ASJ* has published new techniques and technical refinements, as well as key articles on the use of acellular dermal matrices in breast reconstruction, biosynthetic mesh, tranexamic acid administration reducing the risk of postoperative hematoma and seroma, and new insights into patient priorities and emotional outcomes of aesthetic breast reconstruction.^11-,22^

## PREDICTIONS FOR THE FUTURE

*Maurice Nahabedian, MD, FACS*: My predictions for the future are filled with optimism. Plastic surgeons by nature are creative, visual, and artistic and will continue to innovate to provide our patients with the best outcomes.^23^ In many ways, plastic surgery is a regenerative specialty. Although this is especially true with the face, it is also true for the breast. Plastic surgeons will continue to explore the role of autologous fat grafting and ways to improve fat viability and incorporation.^24^ The regenerative potential of fat is becoming more understood, and its role in breast reconstruction and augmentation will one day be a mainstay and possibly reduce the role of artificial prosthetic material.^25^ Artificial intelligence (AI) is rapidly being incorporated into our armamentarium. Plastic surgeons and researchers are continually refining the use of machine-based learning tools like 3-dimensional simulation so potential surgical outcomes can be predicted.^26-28^ AI is also being used to assess soft tissue perfusion using indocyanine green that assists surgeons in reducing the incidence of delayed healing and fat necrosis.^29-33^ The combination of AI and robots continues to be explored for autologous breast reconstruction using deep inferior epigastric perforator and latissimus dorsi flaps.^34,35^ These technological advancements will continue to elevate and expand what is possible for our specialty.

*Nolan S. Karp, MD*: Current implant-based breast reconstruction uses implants designed for breast augmentation. Sometimes these implants work well, and sometimes revisions are required to achieve a good result. In the future, I think we will use computer-aided design/computer-aided manufacturing technology to design custom implants for each individual patient so that an excellent result is achievable in a single stage, when nipple-sparing is possible.

## CONCLUSIONS

In summary, plastic surgery is in exciting times when it comes to aesthetic breast reconstruction. We at *ASJ* are thrilled with the volume of incoming manuscripts and will continue to publish the best of the best. With each passing year, more manuscripts are being submitted that are of high caliber and worthy of publication. Our turnaround times are low, and authors will notice that the time from submission to publication is faster than the scholarly and medical publishing industry standards.^36-38^ We are excited and happy to be affiliated with this premier journal and will continue to promote it nationally and globally.
